# Sildenafil and depression: True or false prophecy

**DOI:** 10.1111/cns.14358

**Published:** 2023-07-14

**Authors:** Hayder M. Al‐Kuraishy, Aseel Awad Alsaidan, Ali I. Al‐Gareeb, Athanasios Alexiou, Marios Papadakis, Gaber El‐Saber Batiha

**Affiliations:** ^1^ Department of Clinical Pharmacology and Medicine College of Medicine, Mustansiriyah University Baghdad Iraq; ^2^ Department of Family and Community Medicine College of Medicine, Jouf University Sakaka Saudi Arabia; ^3^ Department of Science and Engineering Novel Global Community Educational Foundation Hebersham New South Wales Australia; ^4^ AFNP Med, Austria Wien Austria; ^5^ Department of Surgery II University Hospital Witten‐Herdecke, Heusnerstrasse 40, University of Witten‐Herdecke Wuppertal Germany; ^6^ Department of Pharmacology and Therapeutics, Faculty of Veterinary Medicine Damanhour University Damanhour Egypt

Endogenous depression is a behavioral and mental disorder characterized by loss of pleasure, hopelessness, changes in appetite, and sexual dysfunction. The molecular mechanisms of depression are still not well‐known, though reducing synaptic monoamines (dopamine, serotonin, and noradrenaline) could be a possible mechanism.^1^ In addition, chronic inflammatory conditions affect the hypothalamic–pituitary axis (HPA), which affects the neurochemistry of neurotransmitters and the release of hypothalamic and pituitary hormones.^1^ Management of depression by antidepressant agents may induce the development of erectile dysfunction, which may aggravate the propagation of depression.^2^ It has been reported that prolonged treatment with antidepressant agents may trigger the development of erectile dysfunction.^3^ However, atypical antidepressant bupropion which is a dual norepinephrine and dopamine reuptake inhibitor is not associated with the development of erectile dysfunction, a major adverse effect of antidepressant.^4^ Bupropion is also indicated to support smoking cessation, and management of hypoactive sexual desire disorders (HSDD) in women with sexual dysfunction.^5^

Notably, phosphodiesterase inhibitors (PDEIs), like sildenafil which is PDEI type 5, are highly effective in the management of erectile dysfunction.^6^, ^7^ Surprisingly, 18 patients with known cases of endogenous depression on selective serotonin reuptake inhibitors (SSRIs) treated with sildenafil 5 mg/kg for 4 weeks illustrated a remarkable improvement in both erectile function and depressive symptoms according to the Hamilton rating scales for depression.^1^, ^7^ Of note, 68% of patients with erectile dysfunction have different comorbidities including hypertension dyslipidemia, and depression. A previous retrospective study illustrated that sildenafil was effective in the management of erectile dysfunction and associated depression.^1^ A case‐control study confirmed that a daily dose of 5 mg tadalafil a long‐acting PDEI for 2 months improves depressive symptoms considerably.^8^ A recent meta‐analysis including four clinical trials of 270 patients with major depressive disorder (MDD) showed that PDEIs were more effective than SSRIs in the management of MDD.^9^

Therefore, they insist on continuing sildenafil for its beneficial effects in alleviating depressive symptoms.^7^, ^10^ Of interest, low doses of sildenafil improve brain neurotransmitters, mainly serotonin, and noradrenaline, which may decrease the aggravation of depression.^10^, ^11^ Also, sildenafil promotes the synthesis and release of testosterone and oxytocin,^12^ which has antidepressant effects.^13^ Moreover, prolonged treatment with sildenafil improves depressed patients' cognitive functions by improving nitric oxide (NO), which modulates neurotransmitters and HPA action.^14^ Moreover, sildenafil and other PDEIs have potent anti‐inflammatory activity by reducing the expression of pro‐inflammatory signaling pathways such as nuclear factor kappa B (NF‐κB) and high mobility group box 1 (HMGB1) protein.^15^ Inflammation and exaggerated inflammatory signaling pathways are implicated in the pathogenesis of endogenous depression.^16^ Furthermore, a brain‐derived neurotrophic factor (BDNF) which is a neurotrophic factor released from neurons and improves neurogenesis, is highly reduced in depression. It is activated by antidepressant agents, and regarded as a biomarker for response to the effect of antidepressant drugs.^17^ A preclinical study revealed that the administration of sildenafil improves cognitive function in mouse model of Alzheimer's disease by increasing the expression of BDNF.^17^

These observations suggest that sildenafil may have antidepressant effects through the modulation of neurotransmitters and HPA (Figure 1). According to the present findings and speculated sildenafil effects, it could be of value alone or when combined with SSRIs in managing depression. Preclinical and clinical trial studies are recommended in this regard.

**FIGURE 1:**
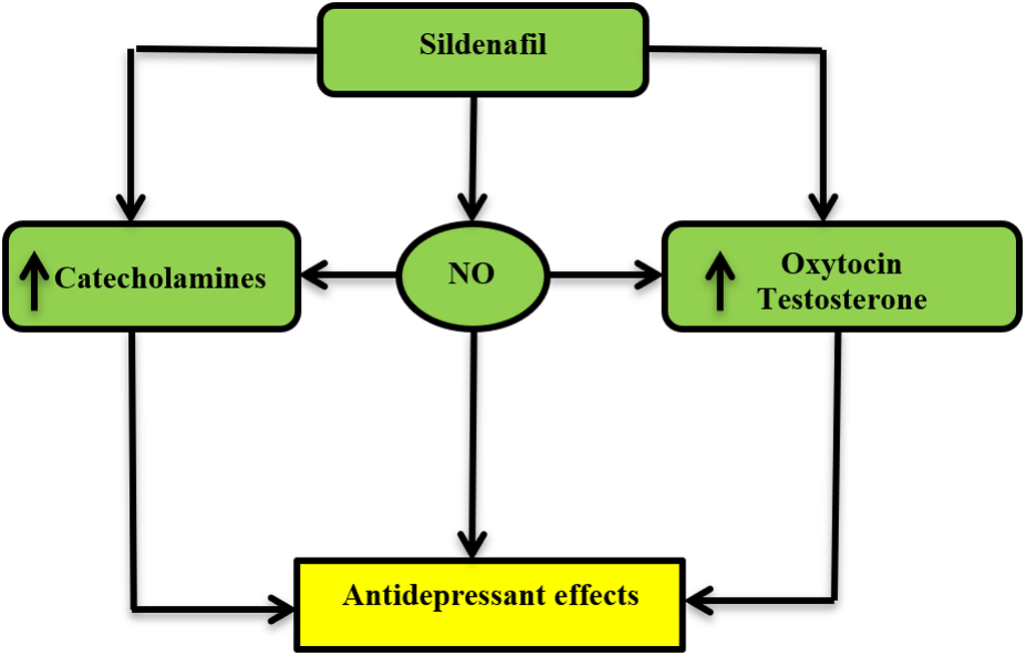
Possible antidepressant role of sildenafil.

## FUNDING INFORMATION

The authors declare that no funds, grants, or other support were received during the preparation of this manuscript.

## AUTHOR CONTRIBUTIONS

Substantial contributions to the conception or design of the work: Hayder M. Al‐Kuraishy and Gaber El‐Saber Batiha. Drafting the work or revising it critically for important intellectual content: All the authors. Final approval of the version to be published: All the authors. Agreement to be accountable for all aspects of the work in ensuring that questions related to the accuracy or integrity of any part of the work are appropriately investigated and resolved: All the authors.

## CONFLICT OF INTEREST STATEMENT

The authors have no relevant financial or non‐financial interests to disclose.

## Data Availability

Data sharing is not applicable to this article as no datasets were generated or analyzed during the current study.
